# Antioxidant Extracts of Three *Russula* Genus Species Express Diverse Biological Activity

**DOI:** 10.3390/molecules25184336

**Published:** 2020-09-22

**Authors:** Marina Kostić, Marija Ivanov, Ângela Fernandes, José Pinela, Ricardo C. Calhelha, Jasmina Glamočlija, Lillian Barros, Isabel C. F. R. Ferreira, Marina Soković, Ana Ćirić

**Affiliations:** 1 Department of Plant Physiology, Institute for Biological Research “Siniša Stanković”-National Institute of Republic of Serbia, University of Belgrade, Bulevar Despota Stefana 142, 11000 Belgrade, Serbia; marina.kostic@ibiss.bg.ac.rs (M.K.); marija.smiljkovic@ibiss.bg.ac.rs (M.I.); jasna@ibiss.bg.ac.rs (J.G.); 2 Centro de Investigação de Montanha (CIMO), Instituto Politécnico de Bragança, Campus de Santa Apolónia, 5300-253 Bragança, Portugal; afeitor@ipb.pt (Â.F.); jpinela@ipb.pt (J.P.); calhelha@ipb.pt (R.C.C.); lillian@ipb.pt (L.B.); iferreira@ipb.pt (I.C.F.R.F.)

**Keywords:** antioxidant activity, *Russula integra*, *Russula rosea*, *Russula nigricans*, antibacterial agents, antibiofilm activity, cytotoxicity, functional ingredients

## Abstract

This study explored the biological properties of three wild growing *Russula* species (*R. integra*, *R. rosea*, *R. nigricans*) from Serbia. Compositional features and antioxidant, antibacterial, antibiofilm, and cytotoxic activities were analyzed. The studied mushroom species were identified as being rich sources of carbohydrates and of low caloric value. Mannitol was the most abundant free sugar and quinic and malic acids the major organic acids detected. The four tocopherol isoforms were found, and polyunsaturated fatty acids were the predominant fat constituents. Regarding phenolic compounds, *P*-hydroxybenzoic and cinnamic acids were identified in the prepared methanolic and ethanolic extracts, which displayed antioxidant activity through the inhibition of thiobarbituric acid reactive substances (TBARS) formation and oxidative hemolysis; the highest activity was attributed to the *R. nigricans* ethanolic extract. This is the first report on the antibacterial and antibiofilm potential of the studied species, with the most promising activity observed towards *Streptococcus* spp. (0.20–0.78 mg/mL as the minimal inhibitory concentration, MIC). The most promising cytotoxic effect was caused by the *R. integra* methanolic extract on non-small cell lung cancer cells (NCI-H460). Therefore, due to the observed in vitro bioactive properties, the studied mushrooms arise as a source of functional ingredients with potential to be used in novel nutraceutical and drug formulations, which can be used in the treatment of various diseases and health conditions.

## 1. Introduction

The profound impact that worldwide globalization and the accompanying lifestyle has on biological systems has recently been re-evaluated since many diseases in humans have been linked to their deleterious effects on human welfare. Moreover, industrialization and agricultural progress have been connected to the occurrence of diverse pollutants in our environment that consequently, once ingested through respiratory and/or digestive systems, may increase the oxidative stress of our cells [1,2]. Along with radiation, cigarette smoke, polluted food, and drastically increased medicine consumption exemplify exogenous factors that can produce reactive oxygen and nitrogen species (RONS). Normally, RONS are produced inside the cells as byproducts during metabolic processes, such as aerobic respiration, fatty acid and toxic compound degradation, as well as infected cell destruction [3,4,5]. Since the balance in the production and removal of RONS shifts towards their overproduction without adequate neutralization, reactive oxidative species accumulate within the cell and disturb cell structures, such as lipids, proteins, and nucleic acids. The resulting deleterious effects place cells into an “oxidative stress” state, which eventually causes modifications and changes of their functions [6]. Nowadays, many diseases, including cancer, diabetes, cardiovascular and inflammatory diseases, neurological disorders, etc., have been related to oxidative stress, which prompts the question how this state may be reduced [5,7]. RONS involved in normal immune defense activity during chronic inflammation, such as a chronic tonsillitis, may cause tissue damage due to their uncontrolled and unbalanced production [8,9].

Consumption of certain foods has emerged as an important factor for maintaining the balance between the production and neutralization of reactive species [10,11]. Furthermore, utilization of numerous commercial and wild-growing mushrooms in the past years proved their potential to be used as so-called nutraceuticals (a food/part of a food that provides health benefits, including the prevention and/or treatment of a disease [12] due to the capacity of several groups of compounds identified in their fruiting bodies to protect cells from oxidative damage [6,13,14]). These include, among many, phenolic compounds, followed by organic acids, as well as vitamins [6].

The genus *Russula* Pers. belonging to the Russulaceae family comprises more than 780 species with a cosmopolitan distribution, making it the second largest genus in the Agaricomycetes class [15]. Most of the species have not been thoroughly studied for their bioactive compounds profile and diverse biological activities. Having in mind the potential importance of mushroom ingredients in maintaining RONS balance in cells, the primary aim of the present study was to evaluate the antioxidant properties of extracts prepared from three *Russula* species: *Russula integra* (L.) Fr., *Russula rosea* Pers., and *Russula nigricans* (Bull.) Fr., growing wild in Serbia. Nevertheless, we investigated the chemical composition, antimicrobial, antibiofilm, and cytotoxic activities of these species as well, as to evaluate their yet unknown biological potential. The presented manuscript provides exclusive data on various aspects of three *Russula* species biological activities, which may expand their utilization as a source of bioactive molecules. 

## 2. Results and Discussion

### 2.1. Chemical Characterization of Russula Species

According to the author’s best knowledge, this is the first study on the chemical characterization of the three different *Russula* spp. fruiting bodies originating from Serbia.

#### 2.1.1. Nutritional Composition of the *Russula* Species

Carbohydrates were the most abundant constituents in all studied mushrooms, followed by proteins, ash and fat (Table 1). A comparison of each nutrient among all three species showed that *R. rosea* had the highest amount of carbohydrates (82.03 ± 0.1 g/100 g dw), followed by *R. nigricans* (75.26 ± 0.4 g/100 g dw) and *R. integra* (72.4 ± 0.3 g/100 g dw). Proteins were present in similar amounts in *R. integra* (21.3 ± 0.5 g/100 g dw) and *R. nigricans* (19.33 ± 0.5 g/100 g dw), while *R. rosea* (12.2 ± 0.01 g/100 g dw) had the lowest content. The studied species were poor in ash and fat compared to the values obtained for carbohydrates and proteins, which coincides with the recently publish data for other wild mushrooms species [16,17]. Energetic values of the samples were in the range of 384.6–393.66 Kcal/100 g dw, indicating that these species represent a very good low-fat food.

#### 2.1.2. Hydrophilic Compounds of the *Russula* Species

The results regarding the hydrophilic compounds (sugars and organic acids) for the studied wild *Russula* mushroom species are presented in Table 1.

Free sugars found in the studied mushrooms were fructose, mannitol, and trehalose. Mannitol was the most abundant sugar in the studied species (Table 1) as has been previously reported for other wild mushroom species [16,18,19,20]. Among all three species, *R. integra* was the one with the lowest content of free sugars (15.2 ± 0.3 g/100 g dw), while the highest amount of sugars was quantified in fruiting bodies of *R. nigricans* (42 ± 1 g/100 g dw). In *R. rosea* fruiting bodies, mannitol was the only sugar detected.

Among the identified organic acids, quinic acid was quantified in a fair amount in the *R. nigricans* sample (11.26 ± 0.01 g/100 g dw), followed by oxalic and fumaric acids in lower amounts; trace amounts were identified in *R. nigricans* (oxalic acid) and *R. rosea* (fumaric acid). Malic acid was detected in all the tested samples, while citric acid was not quantified in any (Table 1). Kouassi et al. (2016) [21] investigated the profile of organic acids in *R. delica*, *R. lepida*, and *R. mustelina* from Côte d’Ivoire and found five different organic acids (oxalic, fumaric, citric, malic, succinic acid). In this study, fumaric acid (4241 ± 30 mg/kg dw, 4183 ± 20 mg/kg dw and 3477 ± 25 mg/kg dw) and malic acid (3207 ± 10, 833 ± 7 and 4230 ± 20 mg/kg dw) were the main found organic acids [21]. In contrast to the previous study, the main organic acids found in the fruiting body of *R. cyanoxantha* originating from Portugal were fumaric and citric acid [22]. Organic acids may have a protective role against various diseases because of their antioxidant activity. They are able to chelate metals or to delocalize the electronic charge coming from free radicals [23]. Due to its antioxidant activity, oxalic acid showed antibacterial activity, while fumaric acid was confirmed to have anti-inflammatory, neuroprotective and antimicrobial properties [24].

#### 2.1.3. Lipophilic Compounds of the *Russula* Species

Lipophilic compounds (fatty acids and tocopherols) were detected in the studied mushrooms (Table 2). The presence of the 28 fatty acids was investigated. In *R. integra*, 21 different fatty acids were detected, while in *R. nigricans*, 20, and in the *R. rosea* sample, only 10 fatty acids. All three tested species contain significant amounts of the following fatty acids: palmitic (C16:0), stearic (C18:0), oleic (C18:1n9c), and linoleic (C18:2n6c), which coincides with the studies observed for other wild-growing mushrooms [25,26,27]. Comparing between all three species, *R. integra* and *R. nigricans* had the highest content of linoleic acid, while *R. rosea* possessed the highest amount of oleic acid (Table 3). Based on the percentage analysis of the saturated (SFA), mono-(MUFA), and polyunsaturated (PUFA) fatty acids, *R. integra* and *R. nigricans* possessed the largest amounts of PUFA while *R. rosea* had the highest percentage of SFA.

For the studied mushrooms, the four tocopherols isoforms (α-, β-, γ-, and δ-tocopherol) were detected (Table 2), with *R. integra* being the one with the highest total tocopherol content (810 ± 3 µg/100 g dw) and with γ-tocopherol as the dominant isoform, which is consistent with the results obtained for *R. sardonia* [18]. Among the other two samples, α-tocopherol was the only isoform present in *R. rosea*, while in *R. nigricans*, α- and δ-tocopherols were detected (Table 2). It was previously established that tocopherols have a great antioxidant potential (inhibiting the production of new free radicals and neutralizing the existing free radicals) [28]. They also can act preventively on the formation of radicals in the membranes and tissues of biological systems, and they can reduce the risk of degenerative diseases associated with oxidative stress [3,28].

In addition, phenolic acids and related compounds (*P*-hydroxybenzoic and cinnamic acid) associated with antioxidant and antimicrobial activities were also performed in methanolic and ethanolic extracts of the *Russula* species. The phenolic acid identified was *P*-hydroxybenzoic acid and also a related compound, cinnamic acid (Table 3). Cinnamic acid was only present in *R. rosea* methanolic and ethanolic extracts (20.0 ± 0.1 and 89 ± 2 µg/g of extract, respectively), and *P*-hydroxybenzoic acid was detected only in the *R. nigricans* ethanolic extract (101 ± 5 µg/g of extract).

Khatua et al. [29] also performed a study in which the phenolic profile of *R. alatoreticula* ethanolic fraction from India was evaluated. Their work shows the presence of pyrogallol as the most dominant compound manifested in the extract followed by cinnamic acid. In samples studied from Côte d’Ivoire, Kouassi et al. [21] evaluated the methanolic extracts, revealing that quercetin, salicylic acid, and tanninol were the main phenolic compounds identified in *R. delica,* while *R. lepida* contained gallic acid, catechin, and protocatechuic acid as the main phenolic compounds; besides these species, they also identified salicylic acid, tanninol, and catechin in *R. mustelina*. The different composition found can be attributed to the different solvents used for the extraction (ethanol and methanol) as well the species and environmental conditions.

### 2.2. Antioxidant Activity of the Russula Species

In the present study, the antioxidant activities of the methanolic and ethanolic extracts of the three *Russula* species were evaluated via its lipid peroxidation inhibition and antihemolytic activity.

The results for the thiobarbituric acid reactive substances (TBARS) assay presented in Table 4 indicate that higher values correspond to lower antioxidant potential. From the obtained results, it may be observed that all the tested samples possess antioxidant activity, though the methanolic and ethanolic extracts of *R. nigricans* were more potent in comparison to the others. The highest lipid peroxidation inhibition was achieved with *R. nigricans* ethanolic extract (IC_50_ = 17 ± 2 µg/mL) whereas *R. integra* methanolic extract demonstrated the lowest antioxidant activity (IC_50_ = 1720 ± 5 µg/mL) in comparison to the other tested samples.

The oxidative hemolysis inhibition assay (OxHLIA) results are presented in Table 4, by the sample concentration required to protect 50% of the erythrocyte population from the hemolytic action caused by the used oxidizing agent for Δ*t* of 10, 30, and 60 min. For the Δ*t* of 10 min, *R. nigricans* methanolic and ethanolic extracts had the lowest IC_50_ values (1.02 ± 0.07 and 9.2 ± 0.9 µg/mL, respectively). However, these extracts did not present an antihemolytic effect for longer time periods. Probably, the antioxidants present in the extracts were depleted very fast, failing to provide protection over time. Moreover, the higher extract concentrations induced a quicker rupture of the membranes. In the case of *R. integra* and *R. nigricans*, the ethanolic extracts were more efficient in protecting the erythrocyte membrane than the extracts prepared with methanol. In fact, *R. nigricans* ethanolic extract was more powerful than trolox for this Δ*t*. Additionally, *R. integra* ethanolic extract was the only one able to protect the erythrocytes population for 30 and 60 min (requiring 69 ± 2 and 139 ± 3 µg/mL, respectively). Both extracts of *R. rosea* did not show antihemolytic activity. This work represents the first contribution on lipid peroxidation inhibition and antihemolytic capacity for the studied species.

There are several reports discussing the antioxidant activity of *R. integra* [30], *R. lepida* [21,31], *R. nigricans* [30,31], *R. delica* [21,25,30,32], *R. vinosa* [30], *R. brevipes* [31], *R. mustelina* [21], and *R. cyanoxantha* [22] extracts although described by different assays, namely DPPH (2,2-diphenyl-1-picryl-hydroxide) radical scavenging activity, ferric reducing power (FRAP), and the β-carotene-linoleate model system. Additionally, Khatua et al. [33] showed that the rusalan (polysaccharide isolated from *R. alatoreticula* basidiocarps) has good antioxidant activity.

### 2.3. Antibacterial Activity of the Russula Species

Methanolic and ethanolic extracts of *R. integra*, *R. rosea*, and *R. nigricans* showed antimicrobial activity against all tested strains but at different levels (Table 5). The minimum inhibitory concentration (MIC) values were between 0.20 and 30.00 mg/mL, while the minimum bactericidal concentration (MBC) was in the range 0.39–60.00 mg/mL (Table 5). The most susceptible strains tested were *M. luteus* (MICs 0.20–3.12 mg/mL), *S. dysgalactiae* (MICs 0.20–6.25 mg/mL), and *S. pseudopneumoniae* (MICs 0.78–3.12 mg/mL) for all tested extracts, while the most resistant strains were *S. aureus*, *S. hominis*, and *S. maltophilia*, with MICs in the range 6.25–30 mg/mL. None of the tested extracts revealed stronger activity than the commercial antibiotics used as a positive control, namely amoxicillin with clavulanic acid and cefixime. According to the author’s best knowledge, this is the first report regarding antibacterial activity done by the microdilution method for all the studied mushroom extracts. Nevertheless, Alves et al. [34] investigated the antibacterial potential of *R. delica* water extract against *S. aureus*, *S. hominis*, *S. pyogenes*, *S. agalactiae*, and *E. faecalis*. The results obtained in this study compared to ours indicated that *R. delica* had better activity only against *S. aureus* and *S. hominis* (MICs 10 mg/mL) [34]. Another study investigated the antibacterial potential of rusalan, and compared to ours, rusalan had better activity against *S. aureus* [33].

### 2.4. Antibiofilm Activity of the Russula Species

*S. aureus* is one of the major human pathogens that causes a wide range of infections. Staphylococcal infections are difficult to treat due to their ability to form biofilms and to secrete enzymes and toxins, which can cause tissue damage [35]. Nowadays, biofilm formation is recognized as an important virulence factor, which provides bacteria with resistance to diverse chemical, physical, and biological antimicrobial agents [36].

**Figure 1:**
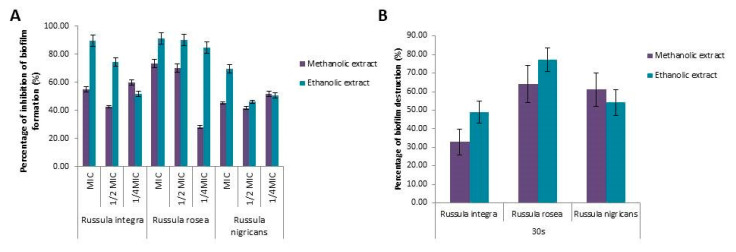
(**A**) Inhibition of *S. aureus* biofilm formation after treatment with MICs and subMICs of *R. integra*, *R. rosea*, and *R. nigricans* extracts. (**B**) Destruction of *S. aureus* 24 h preformed biofilm after 30 s treatment with MBC of *R. integra*, *R. rosea*, and *R. nigricans* extracts. Results are expressed as inhibition percentage, average value of three replicates ± SD.

Ethanolic extracts of all three tested mushrooms showed a stronger potential to inhibit the formation of *S. aureus* biofilms then methanolic. *R. rosea* showed the most promising antibiofilm activity at all tested concentrations (Figure 1A). *R. integra* and *R. rosea* ethanolic extracts inhibit biofilm formation for more than 80% at MICs, while at ½ MIC values, only *R. rosea* ethanolic extract showed activity higher than 80%.

The effects of the 30 s treatment with *Russula* spp. extracts on *S. aureus*-established biofilm was tested. *R. rosea* exhibited better activity than the other two tested species, since it caused biofilm destruction of 64% for methanolic and 77.2% for ethanolic extract (Figure 1B). Both extracts of *R. nigricans* had higher potential for the destruction of the formed biofilm than *R. integra*. *R. nigricans* caused a 61% and 54.2% (methanolic and ethanolic, respectively) biofilm reduction, compared to *R. rosea* causing a reduction of 33% and 48.9% (methanolic and ethanolic, respectively). This is the first report on the antibiofilm activity for all the studied mushroom species; thus, this limits our possibilities of comparing the results obtained herein with the data in the literature.

### 2.5. Cytotoxicity of the Russula Species

The effects of three different mushroom extracts (methanolic and ethanolic) on the growth of four human tumor cell lines (HeLa, HepG2, MCF-7, and NCI-H460) were determined, and the values of GI_50_ are detailed in Table 6. The methanolic extract of *R. integra* revealed activity against all tumor cell lines, except for HepG2. The methanolic extract of *R. rosea* also expressed activity against HepG2 with GI_50_ = 303 ± 8 μg/mL, being less effective against HeLa and NCI-H460 and the least effective against MCF-7. The methanolic extract of *R. nigricans* showed activity only against the HepG2 line (GI_50_ = 372 ± 23 μg/mL). Of the three tested ethanolic extracts, only the *R. integra* ethanolic extract showed an effect against tumor cell lines (MCF-7 and NCI-H460). All the tested extracts of *Russula* species did not exert cytotoxic effects towards non-tumor liver primary cells at the tested concentrations (PLP2; GI_50_ > 400 μg/mL; Table 6). 

In the literature, there are several studies about the cytotoxicity of the compounds isolated from the different species from the genus *Russula*. The study by Zhao et al. [37] demonstrated cytotoxic activity of ergon isolated from *R. cyanoxantha* against HepG2 cells, while Zhang et al. [38] reported antiproliferative activity of lectin isolated from *R. lepida* against HepG2 and MCF-7 cells (IC_50_ = 1.6 and 0.9 μM, respectively).

## 3. Materials and Methods

### 3.1. Mushroom Material

Fruiting bodies of *R. integra* (L) Fr. (Ri-231-2015), *R. nigricans* (Bull.) Fr. (Rn-201-2015), and *R. rosea* Pers. (Rr-211-2015) were collected from Homolje (region of Eastern Serbia) during the October 2015, and authenticated by Jasmina Glamočlija, Principal Research Fellow at the Institute for Biological Research “Siniša Stanković”, National Institute of Republic of Serbia, University of Belgrade. Samples for further research were prepared as previously described [27].

### 3.2. Extract Preparation of the Russula Species

Methanolic and ethanolic extracts from the three *Russula* species were prepared as previously described [27,39]. The extracts were used for determination of phenolic compounds and biological activities.

### 3.3. Chemical Characterization of Russula Species

#### 3.3.1. Nutritional Composition

Proteins, fat, ash, and energy values were determined using procedures approved by AOAC [40]. Carbohydrates were calculated by the difference. The results were expressed in g per 100 g of dry weight (dw) and kcal per 100 g of dw (for energy).

#### 3.3.2. Hydrophilic Compounds

Free sugars were determined by a high-performance liquid chromatography (HPLC) system (Knauer, Smartline system 1000, Berlin, Germany) coupled with a refraction index detector (RI detector, Knauer Smartline 2300). The extraction and whole procedure were performed according to Barros et al. [41]. Compounds identification was made by comparing the relative retention time of sample peaks with standards. Quantification was conducted based on the RI signal response of each standard, using raffinose as the internal standard (IS) method and by using calibration curves obtained from commercial standards for each compound. The results were expressed in g per 100 g of dw.

Following a procedure described by Barros et al. [23], organic acids were determined by ultra-fast liquid chromatography, (UFLC, Shimadzu 20A series, Kyoto, Japan) coupled with a photodiode array detector (PDA). The quantifications were calculated by comparing the area of their peaks, recorded at 215 nm, with the calibration curves obtained from the commercial standards and results were expressed in g per 100 g of dw.

#### 3.3.3. Lipophilic Compounds

Fatty acids were determined, after extraction and transesterification procedures described by Barros et al. [41], using a gas chromatographer (DANI 1000) equipped with a split/splitless injector and a flame ionization detector (GC-FID). The identification of the fatty acids was made by comparing the relative retention times of FAME peaks from samples with standards. Results were recorded and expressed as the relative percentage of each fatty acid.

Following the procedure described by Heleno et al. [28], tocopherols were extracted and determined by HPLC with a fluorescence detector (FP-2020; Jasco, Easton, MD, USA) programmed for excitation at *λ* = 290 nm and emission at *λ* = 330 nm. Compounds were identified by chromatographic comparison with commercial standards and quantification was based on the fluorescence signal response of each standard, using the IS (tocol) method and by using calibration curves obtained from the commercial standards of each compound. The tocopherols content was expressed in µg per 100 g of dw.

#### 3.3.4. Phenolic Compounds Characterization

Phenolic compounds were analyzed with UFLC equipment coupled to a diode array detector (DAD) [39], after dissolving the ethanolic and methanolic extracts in 20% aqueous ethanol and methanol, respectively, at a known concentration. The phenolic acids and related compounds were quantified by comparison of the area of their peaks recorded at 280 nm, with calibration curves obtained from commercial standards. The results were expressed in μg per g of extracts. 

### 3.4. Antioxidant Activity of the Russula Species

#### 3.4.1. Oxidative Hemolysis Inhibition Assay (OxHLIA)

As recommended by Takebayashi et al. [42], erythrocytes were obtained from sheep blood, washed with PBS, and resuspended at 2.8% (*v*/*v*) PBS. Using a flat-bottom 48-well microplate, samples were prepared and incubated, following the Takebayashi et al. [42] procedure. The optical density was measured at 690 nm every 10 min for 1 h. The results were expressed as the delayed time of hemolysis (Δ*t*). The Δ*t* values were then correlated to the different sample concentrations and, from the obtained correlation, the inhibitory concentration (IC_50_ value, µg/mL) of the extracts able to promote a Δ*t* hemolysis delay of 10, 30, and 60 min was calculated [42]. Trolox was the used positive control.

#### 3.4.2. Thiobarbituric Acid Reactive Substances (TBARS) Assay

The lipid peroxidation inhibition was evaluated through the TBARS assay. The antioxidant potential of the methanolic and ethanolic extracts was measured by the decrease in TBARS as described by Kostić et al. [27]. These results were expressed in µg/mL corresponding to the sample concentration providing 50% of the antioxidant activity (IC_50_ value).

### 3.5. Antibacterial and Antibiofilm Activity

#### 3.5.1. Microorganisms: Culture Conditions and Identification

The following Gram-positive and Gram-negative bacteria were used: *Micrococcus luteus*, *Rothia mucilagenosa*, *Streptococcus agalactiae*, *Streptococcus angiosus*, *Streptococcus conselatus*, *Streptococcus dysgalactiae*, *Streptococcus oralis*, *Streptococcus parasanquinis*, *Streptococcus pseudopneumoniae*, *Streptococcus pyogenes*, *Streptococcus salivarius*, *Staphylococcus aureus*, *Staphylococcus hominis*, *Staphylococcus warneri*, *Enterobacter cloacae*, *and Stenotrophomonas maltophilia*. These bacteria were maintained on Blood Agar (Torlak, Belgrade, Serbia). They were obtained from the tonsillar tissue of patients after obtaining informed written consent, at Otorhinolaryngology clinic at Clinical Hospital Center Zvezdara, Belgrade, Serbia. All tested isolates were identified by matrix-assisted laser desorption and ionization time-of-flight (MALDI-TOF) mass spectrometry (VITEK MS bioMerieux, Marcy l’Etiole, France). Tested microorganisms were deposited at the Mycological Laboratory, Department of Plant Physiology, Institute for Biological Research “Siniša Stanković”, National Institute of Republic of Serbia, University of Belgrade.

#### 3.5.2. Microdilution Assay

The antimicrobial activity of methanolic and ethanolic extracts of *Russula* species was determined by the modified microdilution method [43]. The results were presented as minimum inhibitory/bactericidal concentrations (MICs/MBCs). Amoxicillin with clavulanic acid (Hemofarm, Vršac, Serbia) and cefixime (Alkaloid, Skoplje, Macedonia) were used as positive controls.

#### 3.5.3. Inhibition of Biofilm Formation

The ability of methanolic and ethanolic extracts to inhibit biofilm formation was determined with some modification as previously described by Stepanovic et al. [44]. *S. aureus* was selected among the other tested bacteria because of the high MIC/MBC and due to its ability to form oral biofilm. *S. aureus* cells were incubated for 24 h with Tryptic soy broth (TSB) medium (Torlak, Belgrade, Serbia) in 96-well microtiter plates with an adhesive bottom (Spektar, Čačak, Serbia) at 37 °C with MIC and subMIC concentrations of the extracts. After incubation and fixation of the cells, plates were dried and stained using 0.1% crystal violet dye (Bio-Merieux, France). Wells were washed again, and air dried, after which 96% ethanol (Zorka pharm, Šabac, Serbia) was added and the absorbance was read at 570 nm using a plate reader. According to Kostić et al. [27], the percentage of inhibition of biofilm formation was calculated.

*S. aureus* was grown in TSB enriched with 2% glucose in microtiter plates with an adhesive bottom for 24 h at 37 °C. Wells were washed with sterile PBS and the remaining biofilm was treated for 30 s with methanolic and ethanolic extracts obtained from *Russula* species at MBC values. The wells were washed; the biofilm was fixed with methanol, air dried, and stained with crystal violet (0.1%). After dissolving the crystal violet in ethanol, the absorbance was read and according to Smiljković et al. [45], the percentage of biofilm diminishing was calculated. 

### 3.6. Evaluation of the Cytotoxicity in Tumor and Non-Tumor Cells

The cytotoxicity was evaluated in human tumor cell lines (MCF-7, NCI-H460, HCT-15, HeLa, and HepG2) and in a non-tumor liver cell primary culture (PLP2). The methanolic and ethanolic extracts of three different *Russula* species were dissolved in water (8 mg/mL), whereas ellipticine was used as the positive control. All results were expressed as the sample concentration that inhibited 50% of the net cell growth (GI_50_ values, μg/mL) [46].

### 3.7. Statistical Analysis

The statistical analysis was performed through analysis of variance (ANOVA) and Tukey’s HSD test (*p* = 0.05). A two-tailed paired Student’s *t*-test was used for the comparison between two samples (RRM and RRE), and applied to assess the statistical differences (*p* = 0.05). All the analyses were performed using SPSS Statistics software (IBM SPSS Statistics for Windows, Version 23.0. Armonk, NY, USA: IBM Corp.).

## 4. Conclusions

This study constitutes the first report on the chemical composition, antioxidant, antibacterial, and antibiofilm activity of three *Russula* species originating from Serbia. The study indicates that the studied *Russula* species represent a rich source of bioactive molecules with potent in vitro activities, especially for antioxidant activity. In consonance with the investigated chemical composition and biological activities of these mushrooms, they might be further explored in order to be used as nutraceuticals or in functional formulations. Different mechanisms of action involved with specific bioactive compounds present in these species should be further investigated.

## Figures and Tables

**Table 1:** Nutritional value and free sugars and organic acids composition of the studied mushrooms (mean ± SD, *n* = 3).

Constituent	*R. integra*	*R. rosea*	*R. nigricans*
Nutritional Value (g/100 g dw)			
Fat	1.12 ± 0.04 ^b^	0.84 ± 0.01 ^c^	1.70 ± 0.06 ^a^
Protein	21.3 ± 0.5 ^a^	12.2 ± 0.01 ^c^	19.33 ± 0.5 ^b^
Ash	5.19 ± 0.04 ^a^	4.9 ± 0.01 ^b^	3.71 ± 0.08 ^c^
Carbohydrates	72.4 ± 0.3 ^c^	82.03 ± 0.1 ^a^	75.26 ± 0.4 ^b^
**Energy** (Kcal/100 g dw)	**384.9 ± 0.3 ^b^**	**384.6 ± 0.3 ^b^**	**393.66 ± 0.4 ^a^**
Free Sugars (g/100 g dw)			
Fructose	0.347 ± 0.002 ^b^	nd	0.39 ± 0.01 ^a^
Mannitol	14.6 ± 0.3 ^c^	25.8 ± 0.3 ^b^	34 ± 1 ^a^
Trehalose	0.202 ± 0.004 ^b^	nd	7.2 ± 0.5 ^a^
**Total Sugars**	**15.2 ± 0.3 ^c^**	**25.8 ± 0.3 ^b^**	**42 ± 1 ^a^**
Organic Acids (g/100 g dw)			
Oxalic acid	0.110 ± 0.003 ^b^	1.70 ± 0.01 ^a^	tr
Quinic acid	1.56 ± 0.03 ^b^	nd	11.26 ± 0.01 ^a^
Malic acid	1.29 ± 0.02 ^b^	2.06 ± 0.09 ^a^	0.60 ± 0.01 ^c^
Citric acid	nd	nd	nd
Fumaric acid	0.080 ± 0.001 ^a^	tr	0.020 ± 0.001 ^b^
**Total organic acids**	**3.04 ± 0.05 ^c^**	**3.76 ± 0.1 ^b^**	**11.89 ± 0.02 ^a^**

tr—traces; nd—not detected; Different letters in the same row indicate significant differences between the means according to Tukey’s HSD test at *p* = 0.05; The bold indicate the total cumulative values.

**Table 2:** Fatty acids and tocopherols composition of the studied mushrooms (mean ± SD, *n* = 3).

Constituent	*R. integra*	*R. rosea*	*R. nigricans*
**Fatty Acids** (relative %)			
C6:0	nd	0.39 ± 0.02	nd
C8:0	0.12 ± 0.01 ^a^	0.085 ± 0.001 ^b^	0.042 ± 0.001 ^c^
C10:0	0.093 ± 0.008 ^c^	0.197 ± 0.008 ^a^	0.159 ± 0.007 ^b^
C12:0	2.20 ± 0.03 ^a^	nd	0.079 ± 0.05 ^b^
C14:0	2.53 ± 0.04 ^a^	0.61 ± 0.02 ^b^	0.241 ± 0.006 ^c^
C14:1	0.126 ± 0.006	nd	nd
C15:0	1.1 ± 0.1 ^a^	0.33 ± 0.01 ^b^	0.35 ± 0.01 ^b^
C16:0	16.6 ± 0.2 ^a^	12.0 ± 0.3 ^b^	8.8 ± 0.3 ^c^
C16:1	1.55 ± 0.01 ^a^	nd	0.80 ± 0.01 ^b^
C17:0	0.572 ± 0.006 ^b^	0.88 ± 0.03 ^a^	0.127 ± 0.001 ^c^
C18:0	6.57 ± 0.02 ^c^	36.3 ± 0.5 ^a^	7.8 ± 0.3 ^b^
C18:1 n9c	21.85 ± 0.04 ^c^	35.74 ± 0.06 ^a^	27.1 ± 0.1 ^b^
C18:2 n6c	38.2 ± 0.2 ^b^	13.39 ± 0.06 ^c^	52.0 ± 0.7 ^a^
C18:3n3	1.45 ± 0.03 ^a^	nd	0.061 ± 0.001 ^b^
C18:3n6	3.27 ± 0.09	nd	nd
C20:0	1.98 ± 0.01 ^a^	nd	0.210 ± 0.004 ^b^
C20:1	0.082 ± 0.001 ^b^	nd	0.247 ± 0.002 ^a^
C20:2	0.58 ± 0.03 ^a^	nd	0.11 ± 0.01 ^b^
C20:3n3	nd	nd	nd
C20:3n6	0.274 ± 0.001	nd	nd
C20:5n3	nd	nd	nd
C22:0	nd	nd	0.264 ± 0.003
C22:1	0.21 ± 0.01 ^a^	nd	0.16 ± 0.01 ^b^
C22:1n9	nd	nd	nd
C22:2	nd	nd	0.207 ± 0.001
C23:0	0.149 ± 0.006 ^a^	nd	1.17 ± 0.06 ^a^
C24:0	0.425 ± 0.006 ^a^	nd	0.16 ± 0.01 ^b^
C24:1	nd	nd	nd
Total SFA (% of total FA)	32.4 ± 0.3 ^b^	50.9 ± 0.1 ^a^	19.4 ± 0.6 ^c^
Total MUFA (% of total FA)	23.81 ± 0.04 ^c^	35.74 ± 0.06 ^a^	28.3 ± 0.1 ^b^
Total PUFA (% of total FA)	43.8 ± 0.3 ^b^	13.39 ± 0.06 ^c^	52.3 ± 0.7 ^a^
**Tocopherols** (µg/100 g dw)			
α-Tocopherol	13.1 ± 0.4 ^c^	19.5 ± 0.3 ^b^	100 ± 3 ^a^
β-Tocopherol	15.8 ± 0.8	nd	nd
γ-Tocopherol	766 ± 2	nd	nd
δ-Tocopherol	15.7 ± 0.4 ^b^	nd	42 ± 4 ^a^
**Total Tocopherols**	**810** **± 3 ^a^**	**19.5** **± 0.3 ^c^**	**142** **± 1 ^b^**

nd—not detected; Different letters in the same row indicate significant differences between the means according to Tukey’s HSD test at *p* = 0.05; The bold indicate the total cumulative values.

**Table 3:** Phenolic and cinnamic acids composition of the studied mushroom extracts (µg/g of extract) (mean ±SD, *n* = 3).

	*P*-Hydroxybenzoic Acid	Cinnamic Acid
RIM	nd	nd
RIE	nd	nd
RRM	nd	20.0 ± 0.1
RRE	nd	89 ± 2
*t*-test	-	<0.001
RNM	nd	nd
RNE	101 ± 5	nd

nd—not detected; RIM—*R. integra* methanolic extract; RIE—*R. integra* ethanolic extract; RRM—*R. rosea* methanolic extract; RRE—*R. rosea* ethanolic extract; RNM—*R. nigricans* methanolic extract; RNE—*R. nigricans* ethanolic extract. Comparison of means of RRM and RRE was performed with Student’s *t*-test at *p* = 0.05.

**Table 4:** Antioxidant properties of the studied extracts (mean ±SD, *n* = 3).

Extract	OxHLIA (IC_50_, µg/mL)			TBARS (IC_50_, µg/mL)
	Δ*t* 10 min	Δ*t* 30 min	Δ*t* 60 min	
RIM	111 ± 4 ^a^	na	na	1720 ± 5 ^a^
RIE	23 ± 1 ^b^	69 ± 2	139 ± 3	960 ± 22 ^b^
RRM	na	na	na	92 ± 4 ^e^
RRE	na	na	na	116 ± 4 ^d^
RNM	9.1 ± 0.7 ^c^	na	na	120 ± 22 ^c^
RNE	1.02 ± 0.07 ^c^	na	na	17 ± 2 ^f^
Trolox	3.1 ± 0.3	8.1 ± 0.2	20.6 ± 0.7	19.6 ± 0.1

IC_50_—Extract concentration required to protect half of the erythrocyte population for a certain Δ*t* in the OxHLIA assay or corresponding to 50% of antioxidant activity in the TBARS assay; na: no antihaemolytic activity. Different letters in the same column indicate significant differences between the means according to Tukey’s HSD test at *p* = 0.05. RIM—*R. integra* methanolic extract; RIE—*R. integra* ethanolic extract; RRM—*R. rosea* methanolic extract; RRE—*R. rosea* ethanolic extract; RNM—*R. nigricans* methanolic extract; RNE—*R. nigricans* ethanolic extract.

**Table 5:** Antibacterial activity of *Russula* spp. extracts (mg/mL).

Bacteria		RIM	RIE	RRM	RRE	RNM	RNE	Amoxicillin + Clavulanic Acid	Cefixime
*Micrococcus luteus*	MIC	0.78	3.12	0.20	0.39	0.78	3.12	0.0002	0.002
	MBC	1.56	6.25	0.39	0.78	1.56	6.25	0.0004	0.003
*Rothia mucilagenosa*	MIC	3.12	0.78	3.12	0.39	1.56	1.56	0.007	0.002
	MBC	6.25	1.56	6.25	0.78	3.12	3.12	0.014	0.003
*Streptococcus agalactiae*	MIC	3.12	1.56	3.12	0.78	1.56	1.56	0.007	0.002
	MBC	6.25	3.12	6.25	1.56	3.12	3.12	0.014	0.004
*Streptococcus angiosus*	MIC	1.56	3.12	6.25	0.78	1.56	3.12	0.028	0.0002
	MBC	3.12	6.25	12.50	1.56	3.12	6.25	0.056	0.0004
*Streptococcus conselatus*	MIC	3.12	6.25	3.12	6.25	0.78	6.25	0.0002	0.0002
	MBC	6.25	12.50	6.25	12.50	1.56	12.50	0.0004	0.0004
*Streptococcus dysgalactiae*	MIC	3.12	6.25	0.39	0.20	3.12	3.12	0.007	0.0002
	MBC	6.25	12.50	0.78	0.39	6.25	6.25	0.014	0.0004
*Streptococcus oralis*	MIC	0.39	0.78	6.25	3.12	3.12	3.12	0.0004	0.002
	MBC	0.78	1.56	12.50	6.25	6.25	6.25	0.001	0.003
*Streptococcus parasanquinis*	MIC	3.12	6.25	0.78	0.39	1.56	1.56	0.004	0.003
	MBC	6.25	12.50	1.56	0.78	3.12	3.12	0.01	0.006
*Streptococcus pseudopneumoniae*	MIC	0.78	0.78	1.56	0.78	3.12	1.56	0.001	0.013
	MBC	1.56	1.56	3.12	1.56	6.25	3.12	0.002	0.027
*Streptococcus pyogenes*	MIC	0.78	3.12	0.78	3.12	0.39	3.12	0.0004	0.0008
	MBC	1.56	6.25	1.56	6.25	0.78	6.25	0.001	0.004
*Streptococcus salivarius*	MIC	6.25	6.25	3.12	3.12	6.25	6.25	0.01	0.013
	MBC	12.50	12.50	6.25	6.25	12.50	12.50	0.014	0.027
*Staphylococcus aureus*	MIC	12.50	12.50	12.50	12.50	12.50	6.25	0.001	0.003
	MBC	>12.50	>12.50	>12.50	>12.50	>12.50	12.50	0.002	0.006
*Staphylococcus hominis*	MIC	>12.50	7.50	>12.50	>12.50	>12.50	7.50	0.004	0.002
	MBC	>12.50	>12.50	>12.50	>12.50	>12.50	>12.50	0.007	0.003
*Staphylococcus warnerii*	MIC	6.25	6.25	3.12	6.25	6.25	3.12	0.001	0.003
	MBC	12.50	12.50	6.25	12.50	12.50	6.25	0.002	0.006
*Enterobacter cloacae*	MIC	>12.50	3.12	6.25	6.25	3.12	1.56	0.028	0.003
	MBC	>12.50	6.25	12.50	12.50	6.25	3.12	0.056	0.007
*Stenotrophomonas maltophilia*	MIC	>12.50	12.50	>12.50	12.50	12.50	6.25	0.003	0.003
	MBC	>12.50	>12.50	>12.50	>12.50	>12.50	12.5	0.007	0.006

MIC—minimal inhibitory concentration; MBC—minimal bactericidal concentration; RIM—*R. integra* methanolic extract; RIE—*R. integra* ethanolic extract; RRM—*R. rosea* methanolic extract; RRE—*R. rosea* ethanolic extract; RNM—*R. nigricans* methanolic extract; RNE—*R. nigricans* ethanolic extract.

**Table 6:** Cytotoxicity and antitumor activity of the studied samples (GI_50_ values μg/mL, mean ±SD).

	Cytotoxicity to Non-Tumor Cell Line	Cytotoxicity to Tumor Cell Lines			
	PLP2 (porcine liver primary culture)	HeLa (cervical carcinoma)	HepG2 (hepatocellular carcinoma)	MCF-7 (breast carcinoma)	NCI-H460 (non-small cell lung cancer)
RIM	>400	338 ± 13	>400	253 ± 1	236 ± 3
RIE	>400	>400	>400	305 ± 12	281 ± 9
RRM	>400	333 ± 7	303 ± 8	381 ± 16	323 ± 7
RRE	>400	>400	>400	>400	>400
RNM	>400	>400	372 ± 23	>400	>400
RNE	>400	>400	>400	>400	>400
Ellipticine	2.3 ± 0.1	0.91 ± 0.1	1.10 ± 0.09	1.21 ± 0.02	1.03 ± 0.09

GI_50_ values correspond to the sample concentration responsible for 50% inhibition of growth in a primary culture of liver cells-PLP2 or in human tumor cell lines. RIM—*R. integra* methanolic extract; RIE—*R. integra* ethanolic extract; RRM—*R. rosea* methanolic extract; RRE—*R. rosea* ethanolic extract; RNM—*R. nigricans* methanolic extract; RNE—*R. nigricans* ethanolic extract.
